# Extract from the Proceedings of the Institution to Promote Cow-Pox Inoculation, in the Town of Nottingham

**Published:** 1806-03

**Authors:** 


### EXTRACT *from the Proceedings of the Institution to promote* COW-POX INOCULATION, *in the Town of* NOTTINGHAM.

AT a meeting of the Medical Faculty, residing in Nottingham, held October 16, 1805, for the purpose of considering the propriety of their coming forward as a Body to promote some plan for a general Cow-pock Inoculation:

*Resolved.* Ist. That it is our unanimous opinion that the inoculation of the Cow-pock, as discovered by Dr. Jenner, illustrated and practised by himself and others, is highly deserving the patronage and support of all ranks, but more especially of the Medical Faculty, as holding forth, under skilful management, the most efficacious protection from the multiplied evils of that periodical pestilence the natural small-pox, and even promising the entire extermination of that direful contagion, whenever the means can be devised of enforcing a general adoption of it at a very early period of life.

S 2 That

That this has several years been the decided opinion of the greater number now present, appears from the individual though ineffectual support they have given to that practice, both by their example in their own families and among their relations, their reasonings, and their offers of gratuitious inoculation to the poor.

2nd. That as it does not appear the practice of Cow-pock inoculation has hitherto been adopted here in the degree that the individual exertions of the Medical Faculty in its favour would have justified us in expecting, we have judged it expedient THUS TO DECLARE IN A BODY, our unanimous opinion in favour of this practice, and our earnest recommendation that some plan may be adopted to encourage a general inoculation of the Cow-pock among all ranks, and that a permanent charitable establishment be formed, under proper directors, for the sole purpose of extending its benefits to the poor gratuitously.

3rd. That as the extension of contagion by the practice of small-pox inoculation is wholly inconsistent with the object we now have in view, we do hereby engage to do all in our power to dissuade from and discourage that practice.

4th. That three of our number be deputed to wait upon the Mayor with these resolutions, requesting his concurrence in their object, so far as to call a general meeting of the inhabitants of the town, in the hope of obtaining the sanction of our fellow citizens, and their support in conducting a measure that appears to us full of importance.

*Resolved,*—that Dr. Pennington, Mr. Attenburrow, and Mr. Calton, be appointed to wait upon the Mayor for the above purpose.

SIGNED,

| | |
|---|---|
| JOHN STORER, M. D. | THOMAS BIRCH |
| WILLIAM MARSDEN, M. D. | THOMAS HUCKELL |
| CHAR. PENNINGTON, M. D. | THOMAS CALTON |
| JAMES CLARKE, M. D. | ROBERT THOMPSON |
| THOMAS WRIGHT | JOSEPH FLEWITT |
| JOHN WRIGHT | BENJAMIN MADDOCK |
| WARTON PARTRIDGE | SAMUEL MADDOCK |
| JOHN ATTENBURROW | JOHN BIGSBY |
| THOMAS BASNETT | HENRY. OLDKNOW |
| WILLIAM WILLIAMS | RICHARD WING. |

At a general meeting of the inhabitants of the town of Nottingham, convened by the Mayor, held at the Exchange Hall, on Wednesday the 23d day of October, 1805,

1805, to consider of the best means of promoting a plan of General Vaccination, and of forming an establishment for the purpose of extending its benefits to the poorer classes of this populous town,

EDW. SWAN, Esq. Mayor, in the Chair,

*Resolved unanimously*, that this meeting, impressed by the very interesting representation of the gentlemen of the faculty made to the Mayor, entertains the most firm conviction that a permanent establishment for inoculating the poor with the Cow-pock, and for promoting a General Inoculation for the same through all ranks of society within the reach of our influence, is worthy of the patronage and support of the great body of the inhabitants of this manufacturing district, and that a public subscription be solicited to be applied to this purpose.

*Resolved unanimously*, that a small permanent committee be appointed to consist of the following gentlemen, viz. The MAYOR, Dr. STORER, Dr. PENNINGTON, The Rev. CHARLES WYLDE. D. D. The Rev. Mr. ALLIOTT, Mr. ATTENBURROW, and Mr. MADDOCK, who shall be called "The Directors of the Institution for promoting a General Inoculation for the Cow Pock, within the town of Nottingham," who are empowered by this Meeting to devise the best means of attaining the object of this Institution, and who being charged with the power of carrying the same into complete execution, shall direct the application of the funds which the public may think proper to devote to this purpose, and be authorized to elect a Secretary, and to appoint new members of the Institution, whenever it may be necessary in consequence of the death, absence, or resignation of any of the present members.

EDWARD SWANN, MAYOR.

*Resolved*, that the thanks of this meeting be given to the Mayor for calling the same, and for his liberal conduct throughout the whole of the business thereof.

The Directors of the Vaccine Institution trust, that a practice which promises such very considerable individual and general advantages, and which has been recommended by the unanimous voice of all the Medical Faculty, will be universally adopted; the only remaining obstacle, that of trouble and expence to the poor, it is the design of the Institution which they have been appointed to conduct, to obviate. Relying on the benevolent liberality of the wealthy, they have engaged Mr. CALTON, on whose skill and attention they can depend, to inoculate the children of the poor at

 their their own houses, and to inspect the progress of the disease; in the discharge of which trust they have the satisfaction to announce, that he will be regularly assisted by Dr. Clarke, and, if necessary, by the advice of the Medical Gentlemen of the Institution; and they earnestly intreat the poor, on their part, to accept the offer which Mr. Calton will make them, as the means of preserving themselves and their families from a most direful disease,—their children from death, or from disorders which render life burdensome and useless,—and their neighbours from a malignant and destructive contagion.—They beg leave, finally, to protest against the practice of small-pox inoculation; a resource, which, although it produces satisfaction to individual persons, yet at the same time exposes the lives and comfort of many hundreds to imminent danger; and is calculated to disseminate and perpetuate the most deadly pest with which the world has been ever visited.

By Order of the Directors,

*Nottingham, Nov.* 12, 1805. GEO. COLDHAM, Sec.

---

We have now the satisfaction of presenting some report of the progress of this laudable institution by the following extracts of a letter addressed to Dr. Walker; on which he has made a few practical observations, in the form of notes, hoping at the time, he says, that our insertion of them may obtain for Dr. Clarke, as well as himself, interesting communication from some of our ingenious correspondents.

*On the Business of the Royal Jennerian Society,*
Dr. Walker, *Salisbury Square, London.*

Sir,

It is time that I acknowledge the receipt of ichor from you, and return my sincere thanks for your kindness; but I was unwilling to write before our practice in vaccination had advanced sufficiently forward to allow some confidence to our reports and observations.

With the vaccine ichor received from you during Mr. Calton's visit in London, I inoculated a very fine child, in which the progress of the disease was perfectly regular, answering to the description of authors on vaccine-pox, as well as to my observations and experiments in eight cases, in the year 1801, during my residence in Edinburgh. From this child we have perpetuated the disease through four hundred hundred and ten persons; although *we* did not use the second supply of ichor, we were too sensible of its value to suffer it to be lost, and other practitioners were favoured with it.

The progress of the disease was very soon observed to be much retarded,* either by very cold weather, or from much exposure to cold, particularly in debilitated children, which form the majority among the poor of this place; this observation induced us, in fact obliged us, in many instances, to take the ichor for inoculation on the seventh or eighth day, by medical computation; a method of calculation I am sorry to say not sufficiently understood, and which should certainly be explained in every treatise on the disease: from inattention to this circumstance, I have no doubt many controversies have arisen; we are particularly cautious not to take ichor even on these days, (7th or 8th) if any fever, areola, circumscribed induration, or change in the fluid, shall have arisen.

The plan first proposed by Dr. Pearson or Mr. Bryce, (for both claim it) of testing, is that which we follow, but inoculate by one puncture only, and on the sixth day in the other arm; which second inoculation, for so it certainly is, we are seldom refused: it is necessary to remark that the day does not guide us in this test but the state of the vesicle; in most cases the last equals the first in every respect but extent. Each patient is visited four times; on the third visit, which is generally on the tenth day, a purgative of jalap, or jalap and calomel in combination, is prescribed; we were induced to adopt this plan in compliance with popular prejudice.†

None are considered secure but when the scab is complete and presents the dark mahogany colour with a shining smooth surface; but when from the test and one or more inoculations, no effect is produced, they are considered unsusceptible of cow-pox.‡

Whenever the vesicle (a term we employ in contradistinction

* Such retardation is a circumstance of common occurrence when the puncture is made too slightly.

† This compliance I uniformly decline at the Central House, remarking, that the vaccine inoculation has even a purifying effect, superceding the necessity of any *other* purgative or purifier.

‡ I have, in one instance, inoculated a child ten times (literally twenty times; both arms being punctured each time;) before I succeeded in producing any effect. How different is this case from that of Robert Hughes! Vide Medical and Physical Journal, vol. xii. p. 45.

tinction to pustule, the first containing ichor, the last pus) is ruptured,* a reinoculation is performed, exclusive of the test; we do not think this so necessary after the tenth day, if the progress have been regular; but if any doubt arise, even then, or on any other occasion, the subject is re-inoculated.

We have not found any inconvenience to attend inoculation during teething, hooping cough, &c. provided that the fever of these affections be not violent.

We have observed a circumstance which we do not remember ever to have seen remarked; that, if any acute disease occurs during the progress of the vaccine *vesicle*, it is retarded, or rather remains stationary, and the *whole surface* of the vesicle, which is unusually large, is depressed, as the pock in variola from too great exposure to cold, debility, &c. although, in other respects, the vaccine vesicle is perfectly correct; no less than six of these cases have occured in our practice.

Eleven subjects have been inoculated, who were suspected to have had the small-pox when very young; in three of these cases the pock proceeded to maturation, but in two of them the erythema, or areola, was very slight, and without any circumscribed induration; in the remaining eight cases, slight inflammation followed the puncture, and a vesicle arose but soon desquamated.

We have in about ten cases observed a peculiar scab following a regular vesicle, it is formed earlier than the usual scab; but I am quite at a loss for words to describe it, so as to be understood; it is of a greyish brown colour, laminated, considerably elevated above the surface of the cuticle, and about four times the dimensions of the common scab; its edges are ragged, and its surface rough; the persons on whom it is observed have in general been scorbutic. Another irregularity is the scab of an amber colour, which does not rise above the surface of the cuticle; we consider it unsatisfactory; in both these irregularities the first scab has been rubbed off: if they be re-inoculated while these scabs remain, the fresh puncture in four or five days

* In the numbers which it continually falls to my lot to inoculate, it is generally extremely easy to determine by the eye when the effect is complete; but, when the pock has been ruptured, and even almost obliterated, if I can feel, about the tenth day, a degree of hardness and tumefaction about the inoculated part, I find in people of every colour that the protection is complete; and without such inflammation I suspect that the effect is never perfect.

days puts on the same appearance; on the other hand if the scabs have fallen off, the inoculation does not take effect.*

The following is a case of the prophylactic power of the cow-pox.

Charles Judge, eight months, Charlotte Square.

Register, No. 221. *Dec.* 27, 1805.

Has been seven days exposed to the small-pox effluvia, from a child in the same house, since dead of the disease in its malignant form. C. Judge was inoculated this morning (Dec. 27) by Mr. Calton, with fresh fluid ichor from No. 181, (vide Register) by two punctures in each arm.

The mother says this child has laboured under ague, attended with convulsions, for three months; countenance sallow; appears much debilitated.

The father was a sailor, is disabled, and begs his bread with his family from town to town.

Jan. 3, 1806. The inoculated parts present well-formed vesicles.

Jan. 9. The vesicles are very complete specimens of cow-pocks.

Jan. 12. Small papulæ have appeared on the limbs and abdomen, but in greater number on the back, resembling swine (chicken) pock, fevered.

Jan. 14. The apices of the pocks full of serum, some desquamating.

Jan. 17. The pocks desquamating, the scabs on the inoculated parts are complete.

The chicken-pox prevails in the neighbourhood. Two children, one of the same house, the other in the next, are now ill of the small-pox; the parents refused to have them vaccinated.

I shall detail one more case, shewing the possibility of the cow-pox and the small-pox proceeding together.

Elizabeth Hall, eighteen months, Poynton Street.

Register, No. 254. *Dec.* 31, 1805.

Has been eleven days exposed to the effluvia of small-pox, with which her brother and bed-fellow is completely covered, in the distinct form, but innumerable; she was this day (Dec. 31) inoculated by four punctures with fresh fluid from No. 217, (vide Register) and appears in perfect health.

Jan.

---

* All these varieties of appearance in the scab appear to me to have been of the same nature with what is noticed in the beginning of the second paragraph of p. 45, vol. xii. Medical and Physical Journal.

Jan. 5, 1806. Inoculations have taken; much fevered; several small papulæ resembling small-pox appear on the face and breast.

Jan. 9. Has about three hundred full formed, distinct, small variolous pustules in various parts of the body; no fever; the four vesicles of cow-pox are perfectly regular.

Jan. 17. Variolous pustules dried up and falling off in scabs. The vaccine scabs very complete. The small-pox has been so mild as not to interrupt her usual amusements.

I shall not make any remark on these two cases, but shall wait with anxiety for your free and unreserved observations, as it is only by liberal open comment that knowledge can be acquired.

I remain, &c.

JAMES CLARKE,
Physician to the Vaccine Institution.

*Nottingham, Jan.* 19, 1806.

